# A Protective Role of Phenylalanine Ammonia-Lyase from *Astragalus membranaceus* against Saline-Alkali Stress

**DOI:** 10.3390/ijms232415686

**Published:** 2022-12-10

**Authors:** Lijuan Fan, Gongfa Shi, Juan Yang, Guiling Liu, Zhaoqian Niu, Wangbin Ye, Songquan Wu, Ling Wang, Qingjie Guan

**Affiliations:** 1 The College of Landscape Architecture, Northeast Forestry University, 26 Hexing Road, Harbin 150040, China; 2 Key Laboratory of Natural Resources of Changbai Mountain & Functional Molecules, Ministry of Education, Yanbian University, Yanji 133002, China; 3 Key Laboratory of Saline-Alkali Vegetation Ecology Restoration in Oil Field (SAVER), Ministry of Education, Alkali Soil Natural Environmental Science Center (ASNESC), Northeast Forestry University, 26 Hexing Road, Harbin 150040, China

**Keywords:** *Astragalus membranaceus*, phenylalanine ammonialyase, *Nicotiana tabacum*, osmotic stress, gene function

## Abstract

Phenylalanine ammonia-lyase (PAL, E.C.4.3.1.5) catalyzes the benzene propane metabolism and is the most extensively studied enzyme of the phenylpropanoid pathway. However, the role of *PAL* genes in *Astragalus membranaceus*, a non-model plant showing high capability toward abiotic stress, is less studied. Here, we cloned *AmPAL* and found that it encodes a protein that resides in the cytoplasmic membrane. The mRNA of *AmPAL* was strongly induced by NaCl or NaHCO_3_ treatment, especially in the root. Overexpressing *AmPAL* in *Nicotiana tabacum* resulted in higher PAL enzyme activities, lower levels of malondialdehyde (MDA), and better root elongation in the seedlings under stress treatment compared to the control plants. The protective role of *AmPAL* under saline-alkali stress was also observed in 30-day soil-grown plants, which showed higher levels of superoxide dismutase (SOD), proline, and chlorophyll compared to wild-type *N. Tabacum*. Collectively, we provide evidence that *AmPAL* is responsive to multiple abiotic stresses and that manipulating the expression of *AmPAL* can be used to increase the tolerance to adverse environmental factors in plants.

## 1. Introduction

Phenylalanine ammonia-lyase (PAL, E.C.4.3.1.5) catalyzes the deamination of phenylalanine (L-phenylalanine to transcinnamic acid), the first and rate-limiting step of the phenylpropanoid pathway [1]. Typical PALs have a conserved Lyase aromatic domain, which contributs to the regulation of plant growth and development, especially in promoting the accumulation of phenylpropionic acid-like products [2]. In higher plants, the function of *PAL* has been extensively studied in the biosynthesis of various secondary metabolites. Given the essential role of the phenylpropanoid pathway in the synthesis of compounds such as coumarins, lignin, flavonoids, and lignans [3], molecular characterization of *PAL* had been performed in many plant species including *Arabidopsis thaliana* [4], *Oryza sativa* [5], and *Nicotiana tabacum* [6]. Four *PAL* genes (*PAL1-4*) had been identified in the model plant *Arabidopsis* [7]. Knocking-out both *AtPAL1* and *AtPAL2* resulted in enhanced sensitivity to ultraviolet-B light in the double mutant compared to wild-type *Arabidopsis*, indicating a role of *PALs* in response to abiotic stresses. Such a role is well aligned with the observations that the phenylpropanoid metabolism is activated in response to a variety of stress conditions including UV exposure [8], disease [9], and wounding [10,11] and the fact that accumulation of phenylpropanoid metabolites and flavonoids protects plants against adverse environmental factors [12,13]. For instance, *PAL* is a key enzyme involved in plant defense against biotic stress. He Jun et al. found that the expression of *PAL* in rice can improve its resistance to brown planthopper [14].

Saline-alkaline stress greatly inhibits crop growth and poses a significant threat to food security [15]. With a growing population and increasing severity of saline-alkaline stress, it is critical to identify genetic resources with strong adaptability and high tolerance. *Astragalus membranaceus* shows a high-level tolerance to drought [16]. In addition, *A. membranaceus* is a commonly used as an anti-cancer herbal medicine as it can enhance immunity and show multiple protective effects such as anti-tumor, anti-inflammation, anti-aging and anti-oxidation [17]. A transcriptomics study showed that drought-responsive genes are activated in *A. membranaceus* in a stress-dose dependent manner [16]. Drought-induced reprogramming of the gene expression network further led to changes of key metabolisms including an impressive 60-fold increase of proline. In addition, a recent small RNA sequencing study identified 27 cold-responsive microRNAs (miRNAs) under cold stress in *A. membranaceus*, many of which could mediate cold responses by regulating metabolism, redox homeostasis, and hormone signaling [18]. However, saline-alkaline tolerance of *A. membranaceus* remains largely unexplored. Therefore, we selected Astragalus, a traditional Chinese herbal medicine that also shows exceptional resistance to abiotic stresses broad applications in arid and saline-alkali regions. Then, as the alanine Aminotransferase, PAL can open the synthesis pathway of plant secondary metabolites such as flavonoids and lignin. These plant secondary metabolites are closely related to the response of plants to abiotic stress, while the plant PAL gene is related to salinity and alkali. Coercive relationships are rarely reported [19].

We previously cloned a novel PAL gene from *A. membranaceus* (*AmPAL*) by reverse-transcriptase polymerase chain reaction (RT-PCR) and rapid amplification of cDNA ends (RACE) [20]. However, the functional relevance of *AmPAL* remains unknown. To study the potential role *AmPAL* in abiotic stress, we systematically characterized *AmPAL* in this study. We found that *AmPAL* encodes a functional protein and that overexpressing *AmPAL* in *N. tabacum* enhances the tolerance under saline-alkali stress. Thus, *AmPAL* can be used as a candidate gene for generating saline-alkali resistant plants.

## 2. Results

### 2.1. Bioinformatics and Subcellular Localization of AmPAL

**Figure 1:**
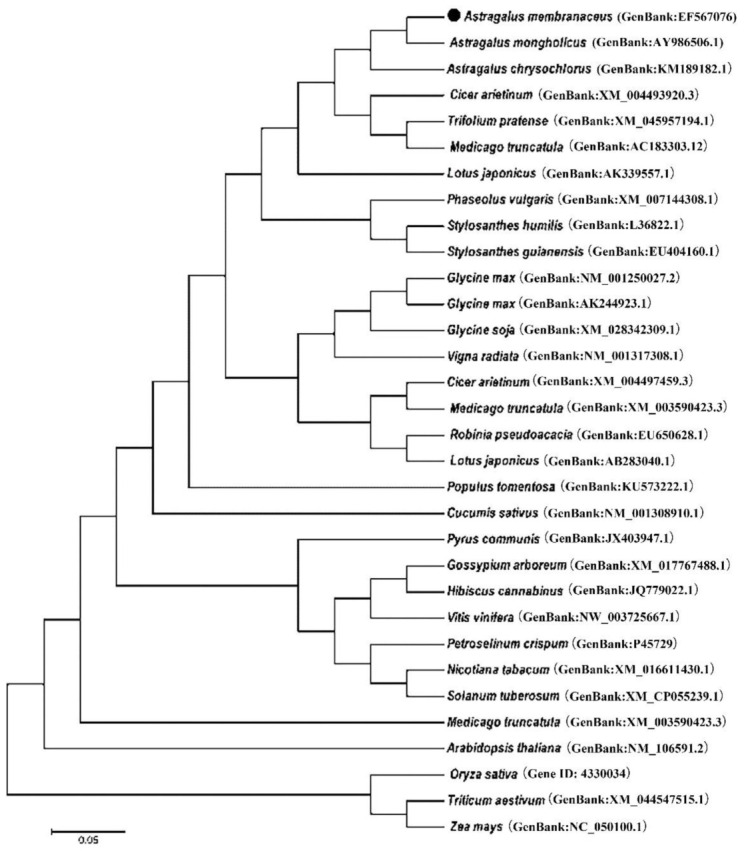
Phylogenetic analysis of PAL protein sequences from different plant species. The *Am*PAL is highlighted (with a dot). The scale bar represents an estimated sequence divergence of 5%. *Astragalus membranaceus* (GenBank:EF567076); *Astragalus mongholicus* (GenBank:AY986506.1); *Astragalus chrysochlorus* (GenBank:KM189182.1); *Cicer arietinum* (GenBank:XM_004493920.3); *Trifolium pratense* (GenBank:XM_045957194.1);*Medicago truncatula* (GenBank:AC183303.12); *Lotus japonicus* (GenBank:AK339557.1); *Phaseolus vulgaris* (GenBank:XM_007144308.1); *Stylosanthes humilis* (GenBank:L36822.1); *Stylosanthes guianensis* (GenBank:EU404160.1); *Glycine max* (GenBank:NM_001250027.2); *Glycine max* (GenBank:AK244923.1); *Glycine soja* (GenBank:XM_028342309.1); Vigna radiata (GenBank:NM_001317308.1); *Cicer arietinum* (GenBank:XM_004497459.3); *Medicago truncatula* (GenBank:XM_003590423.3); *Robinia pseudoacacia* (GenBank:EU650628.1); *Lotus japonicus* (GenBank:AB283040.1); *Populus tomentosa* (GenBank:KU573222.1); *Cucumis sativus* (GenBank:NM_001308910.1); *Pyrus communis* (GenBank:JX403947.1); *Gossypium arboreum* (GenBank:XM_017767488.1); *Hibiscus cannabinus* (GenBank:JQ779022.1); *Vitis vinifera cultivar* (GenBank:NW_003725667.1); *Petroselinum crispum* (GenBank:P45729); *Nicotiana tabacum* (GenBank:XM_016611430.1); *Solanum tuberosum* (GenBank:XM_CP055239.1); *Medicago truncatula* (GenBank:XM_003590423.3); *Arabidopsis thaliana* (GenBank:NM_106591.2); *Oryza sativa* (Gene ID: 4330034); *Triticum aestivum* (GenBank:XM_044547515.1);*Zea mays* (GenBank:NC_050100.1).

The cDNA of *AmPAL* (GenBank accession No. EF567076) was 2650 bp in length with a 2154 bp open reading frame (ORF) that encodes a protein of 718 amino acid residues. Phylogenic analysis showed that *AmPAL* is around 96% homologous to that of *Cicer arietinum*, *Medicago truncatula*, and *Trifolium pretense* (Figure 1). It also showed similarities to PALs from other plant species including *Arabidopsis thaliana*, *Nicotiana tabacum*, and *Oryza sativa*, but to a lesser extent.

**Figure 2:**
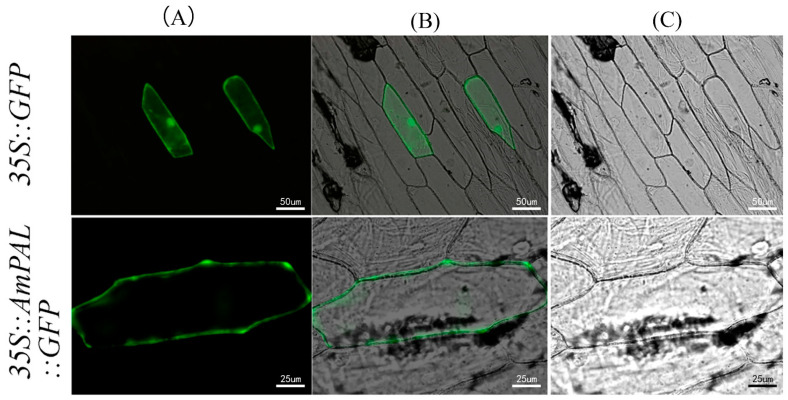
Subcellular localization of *Am*PAL. Upper panel: GFP only as a control; Lower panel: *Am*PAL-GFP fusion protein. The GFP signal (left column) and differential interference contrast microscopy (right column) were merged and shown in the center column. (**A**) GFP: GFP fluorescence signals; (**B**) Merge:GFP fluorescence overlayed on the bright field image; (**C**) Bright Field: image captured under light microscope.

To validate the protein-coding ability of *Am*PAL, the gene was fused with *GFP* and then transiently expressed into onion epidermal cells. As expected, we observed the expression of the fusion protein (Figure 2). In addition, the GFP signal was exclusively confined to the cytoplasmic membrane, indicating that *Am*PAL is localized into the cytoplasmic membrane.

### 2.2. Expression of AmPAL Is Induced by Saline-Alkaline Stress

**Figure 3:**
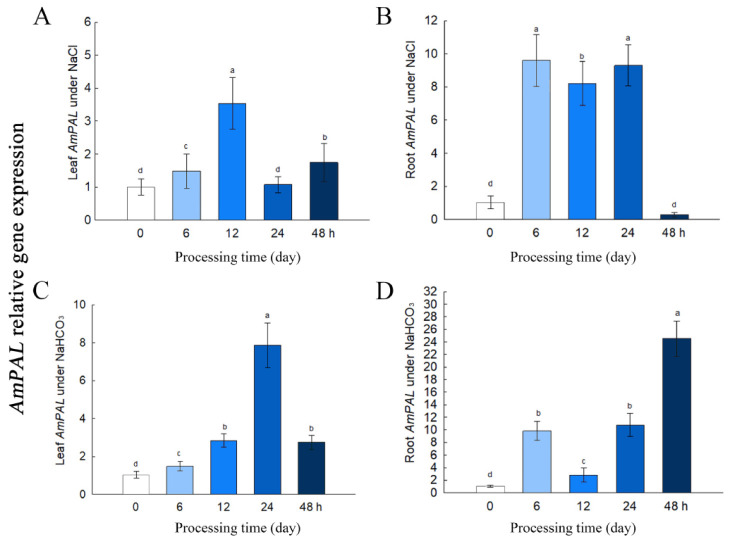
Gene expression of *AmPAL* under 150 mM NaCl or 60 mM NaHCO_3_ for 0, 6, 12, 24, and 48 h. (**A**) Leaf *AmPAL* under NaCl. (**B**) Root *AmPAL* under NaCl. (**C**) Leaf *AmPAL* under NaHCO_3_. (**D**) Root *AmPAL* under NaHCO_3_. Statistical differences are denoted with letters at *p* < 0.01. Data represent mean ±SD from three biological replicates.

To study the stress responsiveness of *AmPAL*, plants were subjected to either 150 mM NaCl (saline stress) or 60 mM NaHCO_3_ (alkali stress) treatment. Time-course gene expression analysis was performed on both the leaf and root tissues (Figure 3). Salt stress activated the expression of *AmPAL* in the leaf, peaking at 12 h after treatment (Figure 3A). The salt-induced expression of *AmPAL* was more pronounced in the root with a 9-fold increase at 6 h after treatment (Figure 3B). Similar to salt, NaHCO_3_ treatment also led to significant increases in *AmPAL* expression in both the leaf (Figure 3C) and root (Figure 3D), with the most significant increase observed in the root at 48 h following the alkali treatment (25-fold increase). The results showed that *AmPAL* was upregulated by the stress of neutral salt and alkaline salt, indicating that *AmPAL* was a functional gene closely related to saline-alkali stress. Higher levels of *AmPAL* in roots suggested that the *AmPAL* might play a major role in roots.

### 2.3. Overexpressing of AmPAL in N. tabacum

We next used a reverse genetics approach to study the functional role of *AmPAL* by overexpressing the gene in *N. tabacum*, a model plant widely used in stress biology. To this end, the *Agrobacterium*-mediated gene transfer method was used to deliver *AmPAL* into the genome of *N. tabacum*. Green buds were observed in the explant (leaf discs) at 21 days on selection and regeneration medium (Figure S1A). Following further growth and root formation (Figure S1B), we obtained healthy plantlets (Figure S1C) that bloomed after 2-month culture in soil (Figure S1D). Integration of *AmPAL* into the *N. tabacum* genome was confirmed by PCR, which revealed five independent lines showing a 2 kb DNA bands as in the positive control (Figure S2A).

**Figure 4:**
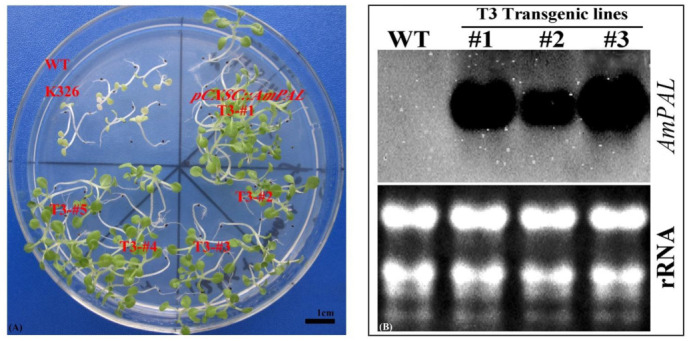
Generation and validation of transgenic *N. tabacum* overexpressing *AmPAL.* (**A**) T_3_ transgenic plants on selective medium. Five independent lines (#1–5) were shown. WT: wild type plants (K326). (**B**) Northern blot analysis of WT and transgenic lines (#1–3). K326: the tobacco variety used in this study; WT: wild-type; pCXSC::*AmPAL*: AmPAL was cloned into the pCXSC vector for expression; T3: the third generation of transgenic plants.

We collected the seeds of the first-generation transgenic plants (T_1_) and further obtained seeds of the third generation (T_3_). As expected, multiple independent lines of the T_3_ seeds geminated well and developed normally on the selective media (50 mg/L Hyg), while the wild-type control (K326) plants died 3 weeks after selection (Figure 4A). Further molecular verification of plant transformation was conducted by Northern blot (Figure 4B), and the results showed that there was a strong signal in the transgenic lines (T3 plants, lines #1–3), and all the three lines could express super-strong transcription under CaMV initiation.

Southern blot analysis of these lines showed that a single copy of *AmPAL* was incorporated into the genome of *N. tabacum* (Figure S2B). We also observed variations in the size of DNA fragments in the Southern blot analysis, indicating non-identical integration sites into the genome.

### 2.4. Impact on Root Growth under Saline-Alkali Stress

**Figure 5:**
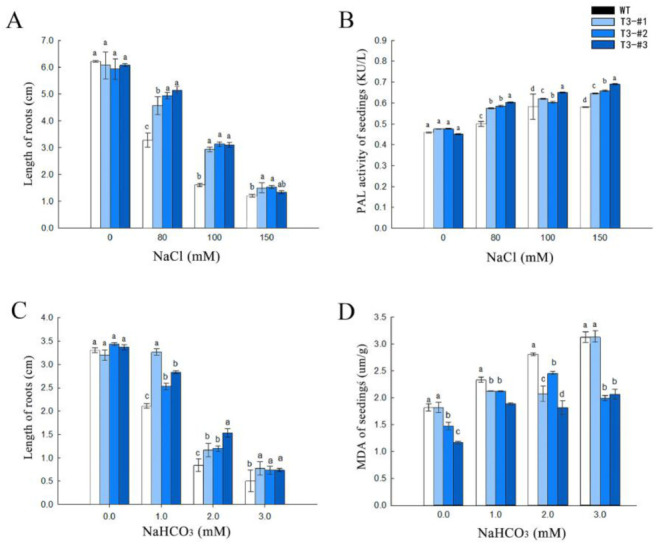
Seedling root growth under either NaCl or NaHCO_3_ treatment. (**A**) Root length under NaCl treatment. (**B**) PAL enzyme activity under NaCl. (**C**) Root length under NaHCO_3_ treatment. (**D**) MDA levels under NaHCO_3_ treatment. Marking: The letters on the bar chart are marked for significance analysis. Where there is one same marking letter, the difference is not significant; where there is different marking letters, the difference is significant. The more different letters are, the more significant they are.

Since root elongation in seedlings is greatly inhibited by saline-alkali stress, we next determined the impact of overexpressing *AmPAL* on root growth by exposing 10-d seedlings to NaCl (0, 80, 100, and 150 mM) or NaHCO_3_ (1, 2, and 3 mM). For both WT and three independent lines of transgenic seedlings, a saline-alkali stress dose-dependent decrease in root growth was observed (Figure 5A,C and Figure S3). However, all three independent transgenic lines showed a significantly longer root length than that of control plants under all stress stimuli tested.

To ascertain the role of *AmPAL* in the enhanced tolerance, we measured the enzyme activity of PAL under salt stress and found that the transgenic plants showed higher PAL activities compared to WT under NaCl treatment (Figure 5B). For NaHCO_3_ treatment, we further quantified the accumulation of MDA and found a dose-dependent increase of MDA in the control plants as the concentration of NaHCO_3_ increased (Figure 5D). We also found lower MDA levels in the transgenic plants compared to the control under NaHCO_3_ treatment. This indicated that overexpressing *AmPAL* gene alleviates NaHCO_3_-induced damages with a lower level of MDA accumulation.

### 2.5. Enhanced Salt Tolerance in Soil-Grown Plants by Overexpressing AmPAL

**Figure 6:**
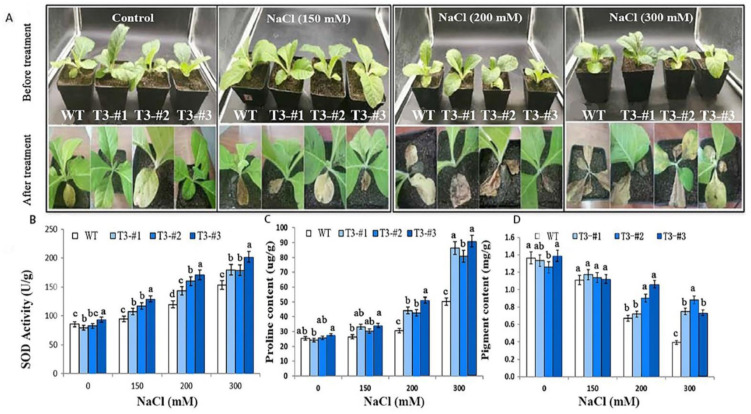
Enhanced salt tolerance in *N. tabacum* overexpressing *AmPAL*. (**A**) Plant phenotypes. The upper and lower panels indicated plants before and after treatment, respectively. One control and three NaCl treatment groups (150, 200, and 300 mM, respectively) were included. For each group, one WT and three transgenic plants (#1–3) were shown. (**B**–**D**): quantification of SOD, proline, and chlorophyll, respectively. Marking: The letters on the bar chart are marked for significance analysis. Where there is one same marking letter, the difference is not significant; where there is different marking letters, the difference is significant. The more different letters are, the more significant they are.

Next, we evaluated the impact of overexpressing *AmPAL* on stress tolerance in 30-day soil-grown plants with an increasing concentration of NaCl (0, 150, 200, and 300 mM). While the transgenic plants were phenotypically similar to that of WT plants under control conditions, they showed higher tolerance to salt stress as green leaves can still be seen in the transgenic plants while the WT wilted completely under 300 mM NaCl for 10 days (Figure 6A). In line with this, quantification of SOD (Figure 6B), proline (Figure 6C), and chlorophyll (Figure 6D) also showed largely comparable levels of these biochemical manifestations under control conditions.

By contrast, much higher/lower levels of these analytes were found in the transgenic plants compared to WT under salt stress (Figure 6A). The difference could be attributed to a higher level of increase/decrease in the transgenic plants under salt stress. For SOD, the WT plants showed 11%, 40%, and 79% increases under 150, 200, and 300 mM NaCl, respectively, while transgenic showed 38%, 83%, and 116% increases under the same treatment regime (Figure 6B). For proline, the transgenic plants and WT showed 3.3- and 1-fold increases under 300 mM NaCl treatment (Figure 6C). For chlorophyll, increasing levels of NaCl caused a sharp decrease in chlorophyll in the WT plants (Figure 6D). However, a slower NaCl-induced chlorophyll decrease was seen in the transgenic plants, resulting in higher levels of chlorophyll in the transgenic plants compared to WT.

The protective role of overexpressing *AmPAL* for chlorophyll under salt was further investigated by quantifying photosystem II photochemical efficiency (the ratio of variable to maximal fluorescence, or Fv/Fm), photochemical quenching coefficient (qP), non-photochemical quenching coefficient (NPQ), and the actual photochemical efficiency (quantum yield, or Qy). The Fv/Fm was around 0.74 for both WT and transgenic plants under control conditions (Figure S4A). While a 40% decrease in Fv/Fm was observed in the WT plants under 300 mM NaCl, a much smaller decrease was seen in the transgenic plants under the same condition (29%, 22%, and 17% for lines #1, 2, and 3, respectively). For NPQ, the WT showed a bell-shaped pattern under increasing strength of salt stress, whilst the transgenic plants exhibited increasing NPQ values as the concentration of NaCl increased (Figure S4B). For both qP and Qy, salt stress resulted in dose-dependent decreases in both WT and transgenic plants (Figure S4C,D). However, the decrease was more pronounced in the WT plants compared to the transgenic plants.

### 2.6. Enhanced Alkali Tolerance in Soil-Grown Plants by Overexpressing AmPAL

**Figure 7:**
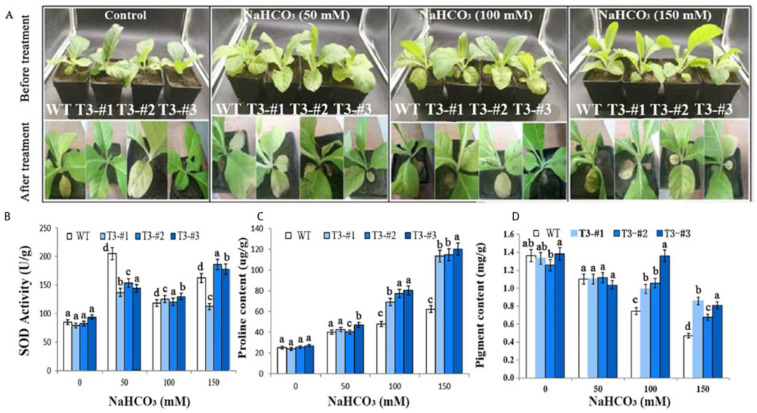
Enhanced alkaline tolerance in *N. tabacum* overexpressing *AmPAL*. (**A**) Plant phenotypes. The upper and lower panels indicated plants before and after treatment, respectively. One control and three NaHCO_3_ treatment groups (50, 100, and 150 mM, respectively) were included. For each group, one WT and three transgenic plants (#1–3) were shown. (**B**–**D**): quantification of SOD, proline, and chlorophyll, respectively. Marking: The letters on the bar chart are marked for significance analysis. Where there is one same marking letter, the difference is not significant; where there is different marking letters, the difference is significant. The more different letters are, the more significant they are.

Using a similar approach, we next investigated the tolerance to alkali stress in soil-grown transgenic plants. An increasing concentration of NaHCO_3_ (0, 50, 100, and 150 mM) caused growth inhibition in both the control and plants overexpressing *AmPAL* with the latter showing better growth performance compared to the former (Figure 7A). Consistent with the salt stress, alkali treatment also led to an increase in SOD activity and proline accumulation but a decrease in chlorophyll (Figure 7B–D). However, the extent of alkali-induced changes was different, resulting in significantly higher levels of proline and chlorophyll in the transgenic plants compared to the control under 150 mM of NaHCO_3_. Further examination of the chlorophyll fluorescence properties in these plants was also performed (Figure S5), which revealed higher Fv/Fm, NPQ, Qy, and qP values in the transgenic plants compared to the control under alkali treatment, especially at high concentrations (e.g., 150 mM of NaHCO_3_).

### 2.7. Enhanced Drought Tolerance in Soil-Grown Plants by Overexpressing AmPAL

**Figure 8:**
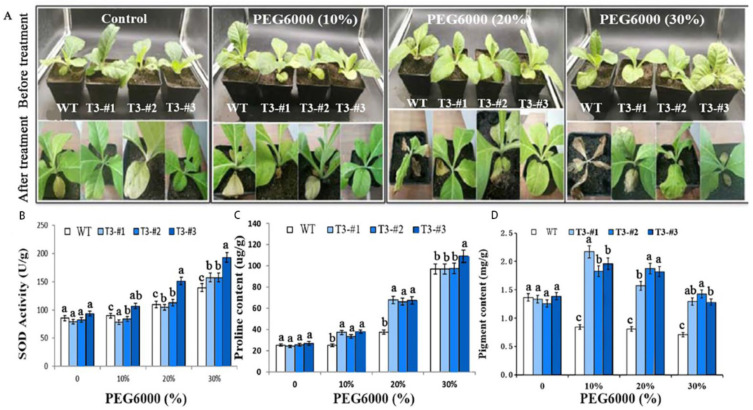
Enhanced drought tolerance in *N. tabacum* overexpressing *AmPAL*. (**A**) Plant phenotypes. The upper and lower panels indicated plants before and after treatment, respectively. One control and three PEG6000 treatment groups (10%, 20%, and 30%, respectively) were included. For each group, one WT and three transgenic plants (#1–3) were shown. (**B**–**D**): quantification of SOD, proline, and chlorophyll, respectively. Marking: The letters on the bar chart are marked for significance analysis. Where there is one same marking letter, the difference is not significant; where there is different marking letters, the difference is significant. The more different letters are, the more significant they are.

Finally, we determined the role of *AmPAL* in drought tolerance by exposing the soil-grown transgenic plants to PEG6000 (0, 10%, 20%, and 30%) for 10 days. Phenotypically, the transgenic plants significantly outperformed the control plants as the former died but the latter survived with 30% PEG6000 (Figure 8A). As expected, much-high levels of SOD, proline, and chlorophyll were observed in the transgenic plants compared to the control (Figure 8B–D). Notably, PEG treatment caused an increase in chlorophyll in the transgenic plant, but a decrease in the WT plants. Higher levels of chlorophyll in the transgenic plants were also consistent with better performance in chlorophyll fluorescence properties (Figure S6).

## 3. Discussions

The phenylpropanoid pathway is one of the most versatile pathways in plants by linking the primary and secondary metabolisms, allowing plants to dynamically modulate the balance between growth/development and response to environmental stresses. Characterization of PAL, the first enzyme in the phenylpropanoid pathway, thus provides an avenue to study and exploit the biological responses to abiotic stresses in plants. Our study of *PAL* from *A. membranaceus*, a non-model plant showing high tolerability against salt-alkali stress, offered multiple lines of evidence of a protective role of *AmPAL*.

The functional relevance of *AmPAL* was first validated by its protein-coding ability and subcellular localization into the plasma membrane. This is in line with previous studies showing that PAL is mostly located in the cytoplasm [21] and other membrane structures such as the chloroplast, leucoplast, mitochondria, peroxisome, and glyoxysome [21,22]. Interestingly, the roles of PAL genes could be dependent on the subcellular localization. The plasma membrane localization of *AmPAL* may have important implications for its function. First, the function of PAL may be comprised under abiotic stress that induces severe damage to the integrity of the membrane. Second, the plasma membrane localization may suggest a fast regulation of PAL activity by environmental stresses. This could be achieved by interacting with other membrane-bound proteins that serve as receptors to environmental factors and small signaling molecules such as reactive oxygen species (ROS). However, the identify of interacting proteins of PAL has not been revealed. Direct regulation of on the enzyme structure and function by ROS also remains elusive.

Second, gene expression analysis showed that *AmPAL* is induced by salt-alkali stress. Compared to the leaf, the root showed the highest levels of *AmPAL* under treatment. This is consistent with previous observations that most *PAL* genes have the highest expression levels in the root [23]. The high abundance of *PAL* in the root had been shown to be required for root development [24]. Southern blot analysis showed that one copy of AmPAL was incorporated into the tobacco genome. In addition, qRT-PCR showed that *PAL* gene was mainly expressed in roots and to a certain extent in leaves. Furthermore, the expression of *AmPAL* in roots and leaves was significantly increased under NaCl and NaHCO_3_ treatment. Collectively, our data supported the key role of *AmPAL* in plant development and further suggested that *PAL* is an abiotic-stress responsive gene in the root. Since the root is the first tissue in sensing and responding to salt-alkali stress, a high level of *PAL* in the root is not surprising. In addition, salt/alkali-induced *PAL* expression also suggested the existence of a gene regulatory network that can sense the stress and initiate the up-regulation of *PAL*. Further investigation is needed to identify the upstream regulatory mechanisms for *PAL* expression.

More importantly, our transgenic work directly demonstrated that overexpressing *AmPAL* enhances the stress tolerance in *N. tabacum*. Physiological/biochemical assessment of three transgenic lines with different mRNA levels of *AmPAL* (Northern blot) showed a correlation between gene expression and phenotype, strongly supporting that the enhanced tolerance under stress is attributable to the overexpression of *AmPAL*. In addition, we observed an increase in the PAL activity under salt stress, further validating that PAL is responsible for increased stress tolerance.

When plants are subjected to environmental stress (including biotic stress and abiotic stress), oxidative stress will be induced, a large amount of reactive oxygen species (ROS) will be accumulated, and the membrane peroxidation of MDA will damage the plant itself. Plants have evolved enzymatic antioxidant defense systems and non-enzymatic antioxidant defense systems to scavenge ROS in the body, and superoxide dismutase (SOD) is the first line of defense to scavenge ROS in plants. MDA content is often used as an important standard for resistance testing of plants under saline-alkali stress. We tested the transgenic plants under multiple stress types including salt, alkaline, and drought, and found the transgenic plants outperformed the control under all stress conditions. Thus, PAL could be a converging point of biological responses to multiple stresses in plants. This hypothesis was supported by our biochemical characterization of MDA, proline, and chlorophyll, where similar results had been found under different environmental stimuli. For instance, a lower MDA level was found in the transgenic plants compared to the control under multiple stresses. MDA targets membrane proteins and causes intramolecular and intermolecular crosslinking, leading to structural and functional damage to the membrane [25]. Thus, overexpressing *AmPAL* alleviated stress damage partially by inhibiting the accumulation of MDA. As MDA is considered an end-point for assessing lipid peroxidation, it is likely that PAL-mediated regulation on MDA is a common pathway toward multiple stresses. Previous studies had also demonstrated a role of PAL in the response to distinct environmental factors including chilling responses in banana [26], defense against *Phytophthora sojae* infection in soybean [27], resistance to fungal pathogens in the grass *Brachypodium* [28], response to phosphate deficiency in rice [29], and wounding responses in the model liverwort *Marchantia polymorpha* [30]. These studies correlated well with the identification of multiple cis-elements in the promoter region of the *PAL* gene [31], highlighting the multifaceted nature of its function. Nonetheless, we also note different responses under distinct treatment schemes. For instance, the directionality of change in chlorophyll was different between PEG and other treatments. In addition, NaCl is a neutral salt and does not change the pH value of the environment, while NaHCO_3_ increases the pH value [32]. Therefore, phenotypical differences between NaCl and NaHCO3 treatment could be explained by the pH difference.

Here, we quantify the overall plant fitness, photosynthesis, chlorophyll, MDA (for lipid peroxidation), and SOD (redox regulation) for understanding the biological responses to stress in both the control and transgenic plants. These could be secondary effects of overexpressing *AmPAL*. The immediate impact of *AmPAL* warrants future studies to elucidate the underlying molecular mechanisms. Because *PAL* is the first enzyme in the phenylpropanoid biosynthesis pathway, its overexpression is expected to lead to an increased level of secondary metabolites. Indeed, a higher level accumulation of several allelopathic phenolics had been found in the root of *Rehmannia glutinosa* overexpressing *RgPAL*, which promoted the replating disease development [32]. Another study showed that overexpressing rice (*Oryza sativa* L.) *PAL8* enhances the resistance to brown planthopper via modulating biosynthesis of lignin and salicylic acid [33]. PAL proteins had also been shown to interact with Kelch repeat F-box protein (KFB), which mediates the protein turnover through the ubiquitin-26S proteasome pathway [34]. How overexpressing *AmPAL* impacts its interaction with other proteins and post-translational regulation had not been determined. Characterization of such interactions is crucial in elucidating the molecular mode of actions among different proteins and the crosstalk between pathways. For instance, the phenylpropanoid biosynthesis pathway (catalyzed by PALs) had been linked to the glucosinolate pathway via the transcriptional regulation of *KFB* genes [35]. Another protein that interacts with PAL is Fe(II)/2-oxoglutarate dependent dioxygenase (SRG1), which stabilizes the protein of PAL in inhibiting cadmium accumulation [36]. In addition, multiple-level and sophisticated regulatory mechanisms exist in controlling the mRNA, protein, and activity of PAL [37], it is easy to imagine that overexpressing *AmPAL* can bring about perturbation to the fine-tuned regulatory network and thus alter the overall phenotype in response to stress.

Research on abiotic stress responses in plants had been aided by the development of novel large-scale omics techniques including transcriptomics, proteomics, and metabolomics [38,39,40]. Application of these approaches would allow the discovery of new molecules and pathways that are significantly changed in transgenic plants overexpressing *PAL*. Since our control and transgenic plants overexpressing *AmPAL* show similar phenotypes under normal conditions, similar molecular phenotypes (gene expression, protein abundance, and metabolite levels) will be expected. Thus, comparative omics studies would be best performed in plants under various abiotic stimuli. Future studies using these techniques will undoubtfully improve our understanding of stress biology and generate new insights in plant engineering for an ever-changing environment.

## 4. Conclusions

We conclude that a *PAL* gene from *Astragalus membranaceus* encodes a functional phenylalanine ammonia-lyase and that the overexpressing *AmPAL* in *N. Tabacum* enhances the tolerance under abiotic stresses. The results presented here are promising in the use of *AmPAL* for plant engineering. Further work is required to better understand the immediate impact of overexpressing *AmPAL* as common end-point physiological parameters (e.g., MDA, proline, chlorophyll) were used in this study. These works would reveal the complicated molecular network governing the function of PAL on one hand and provide better safety guides for genetic engineering of plants on the other.

## 5. Materials and Methods

### 5.1. Plant Materials and Reagents

*Astragalus membranaceus* and *Nicotiana tabacum* K326 were kept at the Agricultural College of Yanbian University. High-fidelity EX-Taq DNA Polymerase, pMD18-T Vector, T4 DNA ligase, *Xcm I* restriction endonuclease, and reverse transcription (RT) kits were purchased from TAKARA Biotechnology (Dalian, China). Gel extraction kit was purchased from MBI (Shanghai, China). Digoxigenin, kanamycin, and hygromycin were purchased from Sigma (Beijing, China). Trizol was purchased from Invitrogen (Shanghai, China). A plasmid extraction kit was purchased from TIANGEN (Bejing, China). *pCXSN* [41], pBI121-MCS-GFP, pMD18-T-PAL plasmid DNA [20], *Escherichia coli* (JM109), and competent cells of *Agrobacterium* (EHA105) were made and kept at our laboratory.

### 5.2. Bioinformatics

Phylogenetic analysis was performed using Mega (v 5.10) with the neighbor-joining method. The Kimura 2-parameter substitution model was selected, and the bootstrap number was 1000. Default settings were used for other parameters.

### 5.3. Subcellular Localization

The coding gene of *AmPAL* was amplified from the pMD18-T::*AmPAL* plasmid [20] using 5′-ggtaccatggagggagaaggagccaat-3′ (forward primer with the *Kpn I* site underlined) and 5′-actagtagaaattggaagaggagcacc-3′ (reverse primer with the *Spe I* site underlined). The PCR product was subcloned into PBI121, and the resulting PBI121-*AmPAL::GFP* was delivered into onion epidermal cells as described before [42,43]. After culture in the dark for 24 h, GFP (green fluorescent protein) fluorescence was observed using a confocal microscope (Olympus, Bejing, China).

### 5.4. Gene Expression Analysis

*A. membranaceus* was cultured in the Hough medium for 21 d and then exposed to 150 mM NaCl or 60 mM NaHCO_3_ for 0, 6, 12, 24, and 48 h. Root and leaf samples were harvested and stored at −80 °C until use. Total RNA was extracted using the Trizol method, and 1 μg RNA was used to synthesize cDNA. For quantitative reverse-transcription (qRT)-PCR, 10 ng/μL cDNA was mixed with the Brilliant III SYBR Green qPCR reagent (Agilent), followed by thermal cycling of 95 °C for 30 s, 58 °C for 30 s, and 72 °C for 30 s. Gene-specific primers were 5′-ggtttcggtgctacttccca-3′ and 5′-agctttggagttaggtcggc-3′. *Actin 1* (forward: 5′-cttcataggaatggaagctgcgggta-3′; and reverse: 5′-cgaccaccttgatcttcatgctgcta-3′) was used as a control. PCR reactions were performed on a MxPro-Mx3000P system. Relative expression was calculated using the 2^−ΔΔCt^ method, the equation is as follows.

$$\Delta C_{t}=C_{t\left(AmPAL\right)}-C_{t\left(\left(Actin1\right)\right)}\;\Delta \Delta C_{t}=C_{t\left(AmPAL\;\mathrm{stress}\right)}-C_{t\left(AmPAL\;\mathrm{control}\right)}$$

### 5.5. Vector Construction and Plant Transformation

First, the open reading frame (ORF) of *AmPAL* was amplified by PCR (forward: 5′-ggtaccatggagggagaaggag ccaat-3; and reverse: 5′-ctaagaaattggaagaggagcac-3′) from the pMD18-T*::AmPAL* plasmid. Next, the ORF was subcloned into pCXSN (pretreated with *XcmI*). Electroporation was used to introduce the *pCXSN::AmPAL* construct into *Agrobacterium* (EHA105).

The *Agrobacterium*-mediated method was used for transformation [44]. A positive clone of EHA105 was cultured in YEP (supplemented with 100 mg/L rifampicin and 50 mg/L kanamycin) to OD_600_ of 0.6. Bacteria were collected and diluted 3-fold with the Murashige-Skoog (MS) medium. Seeds of *N. tabacum* were sterilized in 70% ethanol for 30 s and then 1% sodium hypochlorite for 20 min. Leaves of 20-d aseptic seedlings were cut into 0.5 cm^2^ leaf discs, which were then immersed in the bacterial culture for 5 to 10 min and then cultured in MS containing 30% sugar and 0.8% agar at 26 °C in the dark for 3 d. Subsequent selection and regeneration was performed using MS containing 0.5 mg/L 6-BA (6-Benzylaminopurine), 0.1 mg/L NNA (1-Naphthaleneacetic acid), and 50 mg/L hygromycin (Hyg). The obtained seedlings were then further cultured on ½ MS without hormone. Transgenic plants were screened for three successive generations.

### 5.6. Identification of Transgenic Plants

For PCR identification, leaf DNA was extracted using the CTAB method [45]. PCR was then performed to verify gene integration into the transformed plants. Southern blot was performed as previously described [46]. Briefly, 10 μg of genomic DNA was digested with *BamHI* overnight and then separated by 1% agarose gel electrophoresis. Following transfer to a membrane and fixation with UV light, a digoxigenin (DIG)-labeled probe was applied for hybridization. The *pMD18-T::AmPAL* plasmid was used as a positive control.

Northern blot was performed to examine the transcription of *AmPAL* in transgenic plants [46]. Total RNA from WT (K326) and three transgenic lines (T_3_, #1–3) were first extracted with Trizol and then denatured at 65 °C for 10 min. After gel electrophoresis, transferring (to a Hybond-N+ nylon membrane), and cross-linking (by UV irradiation), hybridization with DIG-*AmPAL* was performed at 50 °C for 12 h. The northern blot signal was quantified with the CDP-Star reagent.

### 5.7. Root Growth, PAL Activity, and MDA of Seedlings

Seeds of WT and three independent transgenic lines (T3, #1–3) were sterilized and germinated on ½ MS for 10 d. Seedlings were then transferred to ½ MS supplemented with NaCl (0, 80, 100, and 150 mM) or NaHCO_3_ (0, 1, 2 and 3 mM). Further culture was performed for 21 days for NaCl treatment and 15 days for NaHCO_3_ treatment. Culture conditions were 25 °C with a 16 h/8 h (light/dark) photoperiod. Root length was then determined. PAL activity was evaluated using a PAL-ELISA kit per the manufacturer’s instruction (Boxbio, Bejing, China). MDA (malondialdehyde) was quantified as reported [47].

### 5.8. Abiotic Stress in Soil-Grown Plants

Seedlings were grown normally in soil (Pindstrup, SHANGHAI, CHINA) for 30 days. For salt treatment, plants were treated with 150, 200, or 300 mM NaCl. For alkali stress, plants were treated with 50, 100, or 150 mM NaHCO_3_. For drought, plants were treated with 10%, 20%, or 30% of PEG6000. Control plants were mock-treated with H_2_O. All treatments were performed for 10 days, and 40-day-old plants were used for the following physiology measurements: SOD (superoxide dismutase), proline, chlorophyll, and chlorophyll fluorescence.

The MDA assay was performed using the thiobarbituric acid (TBA) method as described [47]. Briefly, 0.5 g of samples were pulverized in liquid nitrogen. After adding 1 mL of TBA and 1 mL of trichloroacetic acid and incubation in boiling water for 15 min, the absorbance of was measured at 532 nm (A_OD532_) and 600 nm (A_OD600_). A_OD600_ was subtracted from A_OD532_ to correct non-specific absorption.

The proline content was determined by the ninhydrin-based colorimetric method [48]. For each sample, 0.5 g of fresh material was weighted for extraction (2 mL of glacial acetic acid and 2 mL of 2% acidic ninhydrin). The mixture was extracted with 4 mL of toluene, and the supernatant was used for quantification at an absorbance of 520 nm. Quantification was performed against a standard curve.

For chlorophyll assay, 1.0 g of leaves were ground in 3 mL of 95% ethanol using a mortar and a pestle. The extract was filtered and diluted to 50 mL. The absorptance was recorded at 645, 663, and 470 nm. Calculation of subclasses of chlorophyll was performed as described [49].

Chlorophyll fluorescence data were obtained using a FluorCam fluorescence imaging system (Photon Systems Instruments, Drásov, Czech theUSA) accordingly to published protocols [50]. Plants were first dark-adapted for 30 min. The following four parameters were determined: maximum quantum yield of PSII photochemistry, the actual quantum yield of photosystem II, and non-photochemical quenching, steady-state non-photochemical quenching.

### 5.9. Statistical Analysis

One-way or two-way ANOVA was used for multiple group comparisons. Statistical difference at an alpha level of 0.05 was denoted by different letters. Data in all bar graphs represent mean and standard deviation. Three biological replicates were conducted for all assays.

## Data Availability

Data from this study are available from the corresponding author upon reasonable request.

## Supplementary Materials

The following supporting information can be downloaded at: https://www.mdpi.com/article/10.3390/ijms232415686/s1.
