# Supplementary figures and images for: A Protective Role of Phenylalanine Ammonia-Lyase from Astragalus membranaceus against Saline-Alkali Stress
Source: Int J Mol Sci. 2022 Dec 10;23(24):15686. doi: 10.3390/ijms232415686 (PMC9779599; doi:10.3390/ijms232415686)

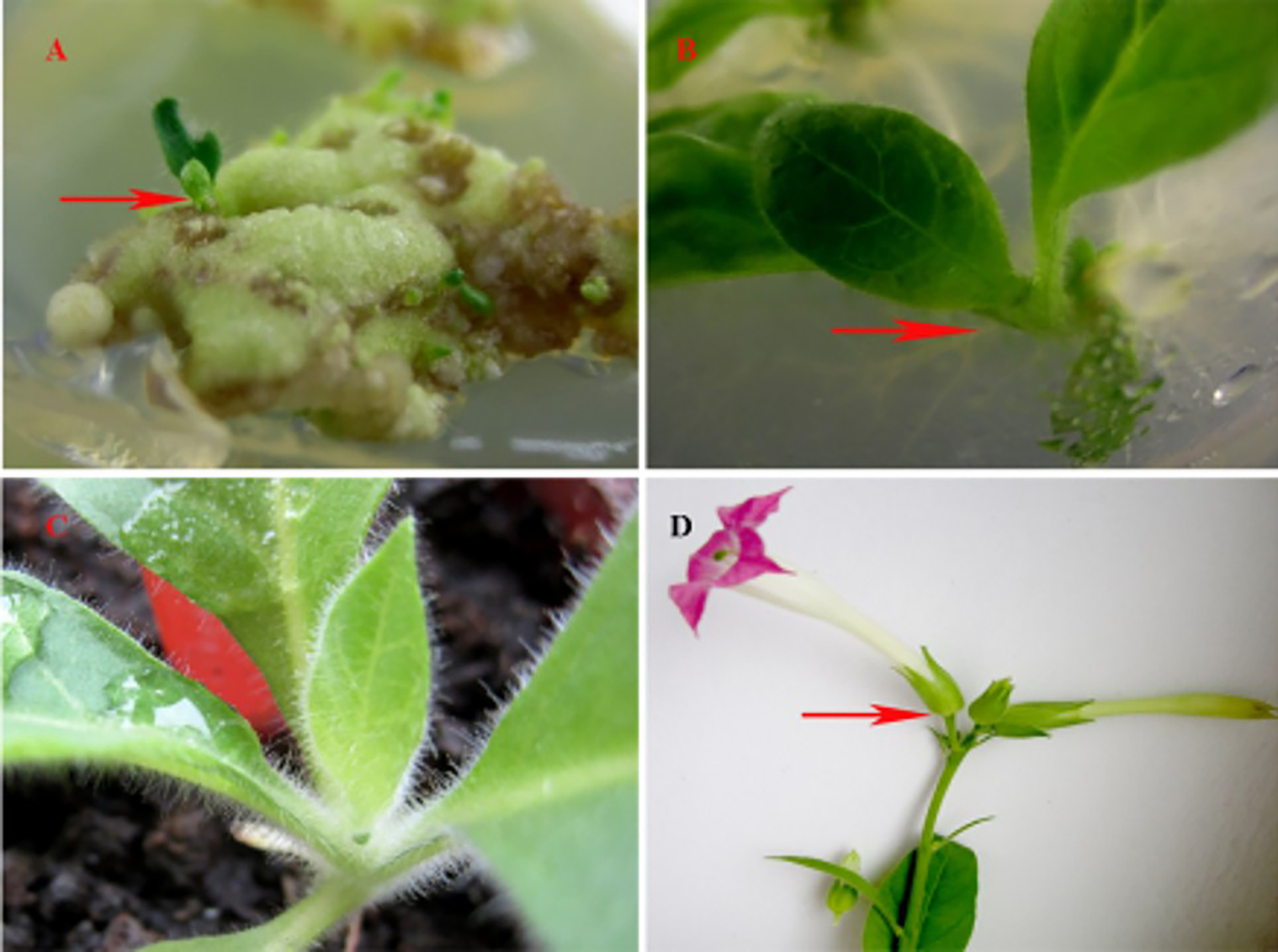

Supplement: Supplementary file 1 [file ijms-23-15686-s001.zip › S1.jpg]

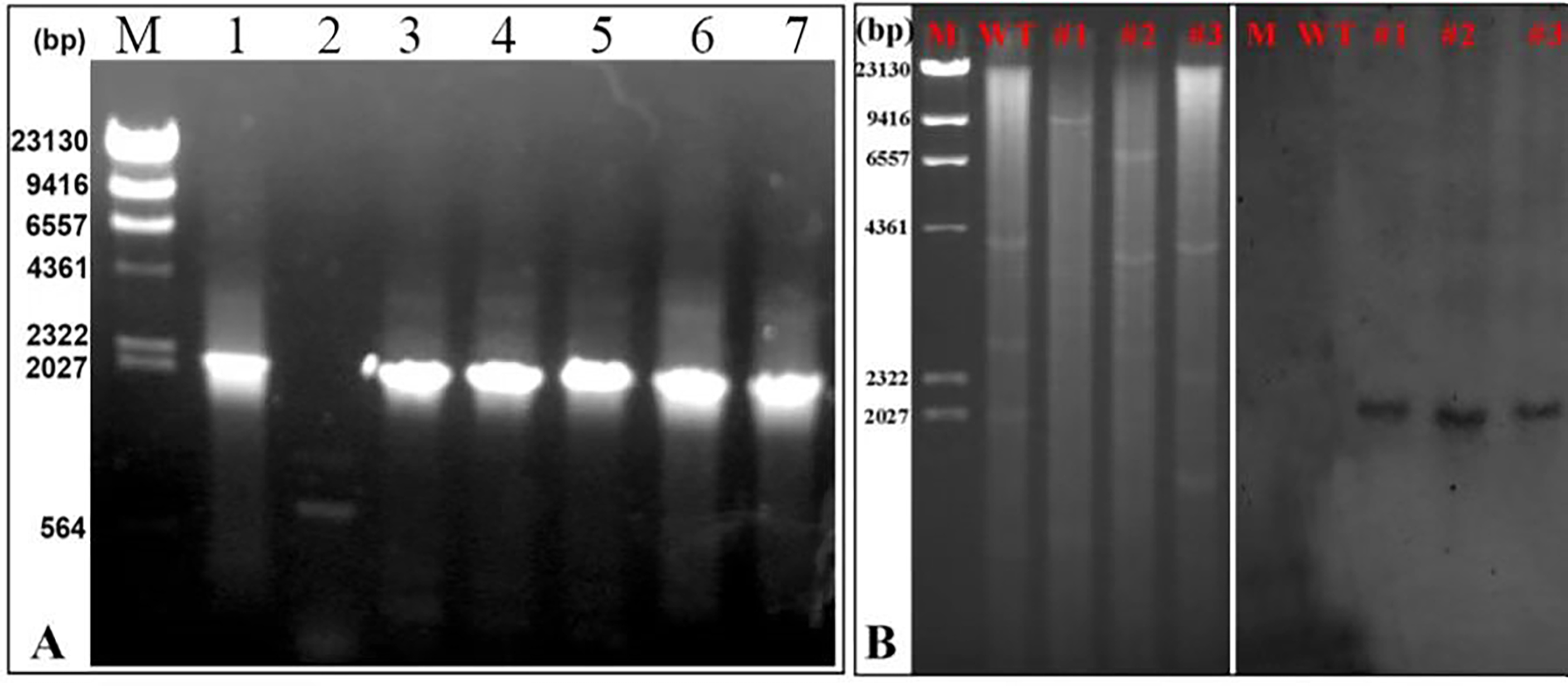

Supplement: Supplementary file 1 [file ijms-23-15686-s001.zip › S2.jpg]

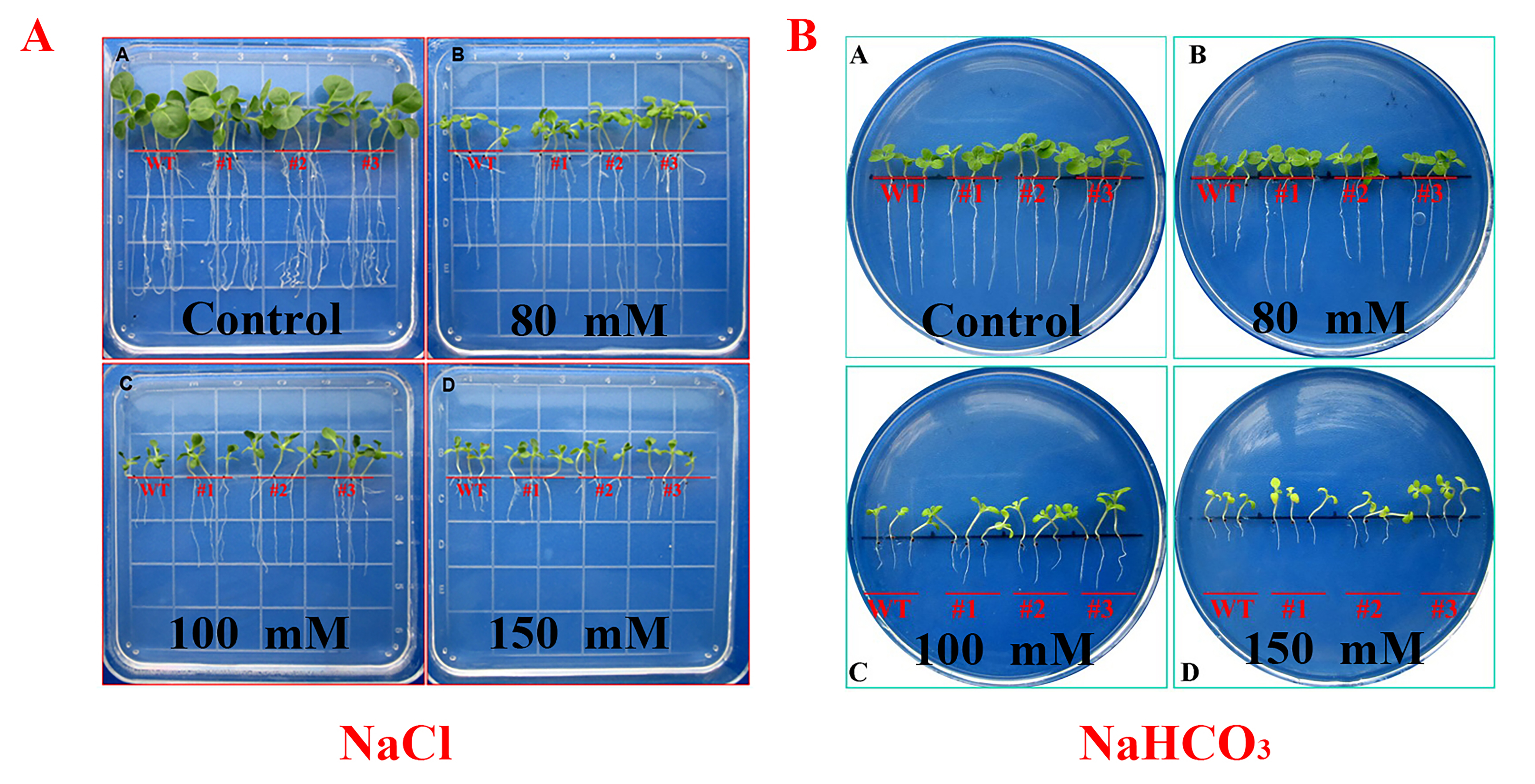

Supplement: Supplementary file 1 [file ijms-23-15686-s001.zip › S3.jpg]

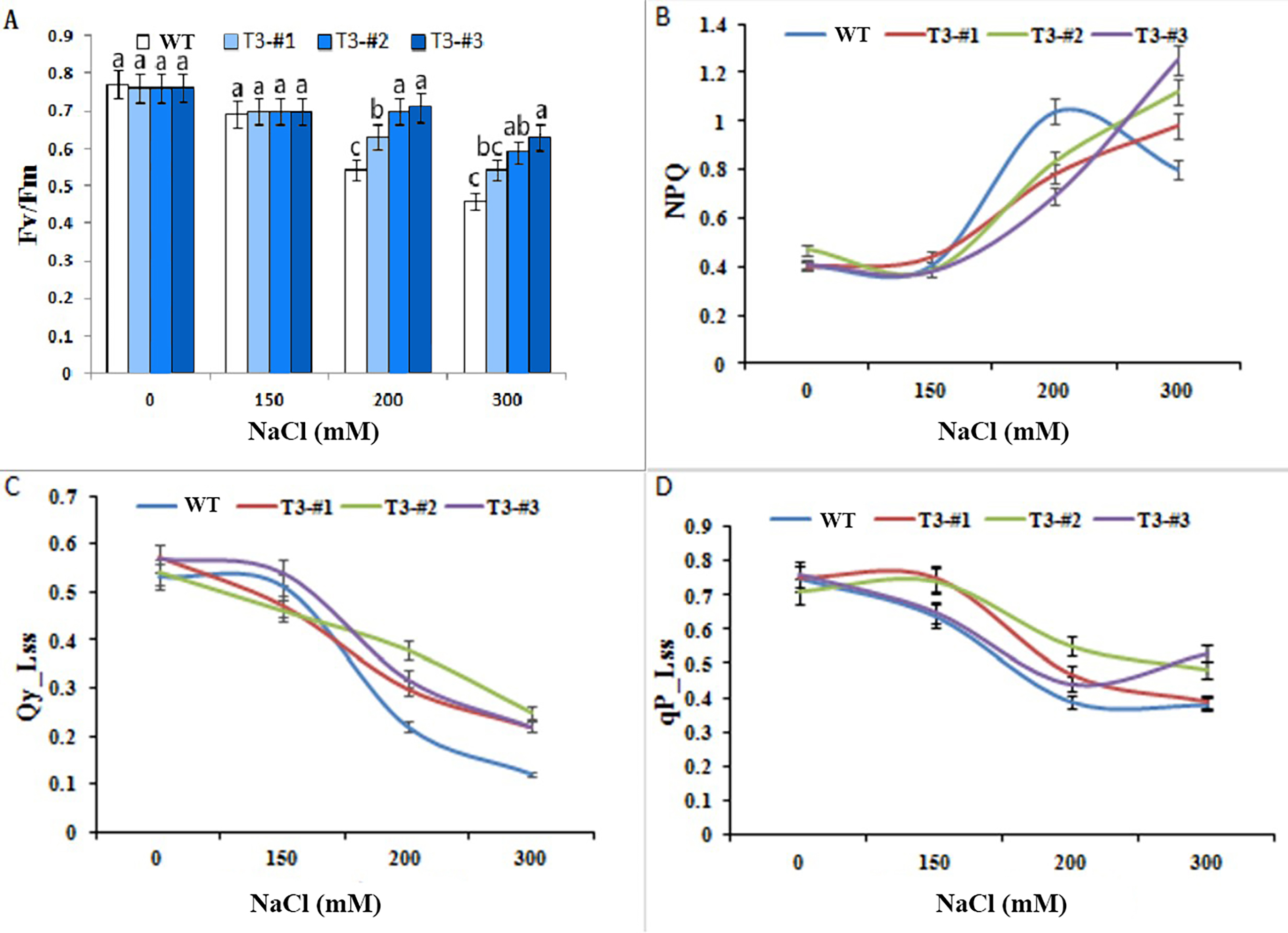

Supplement: Supplementary file 1 [file ijms-23-15686-s001.zip › S4 .jpg]

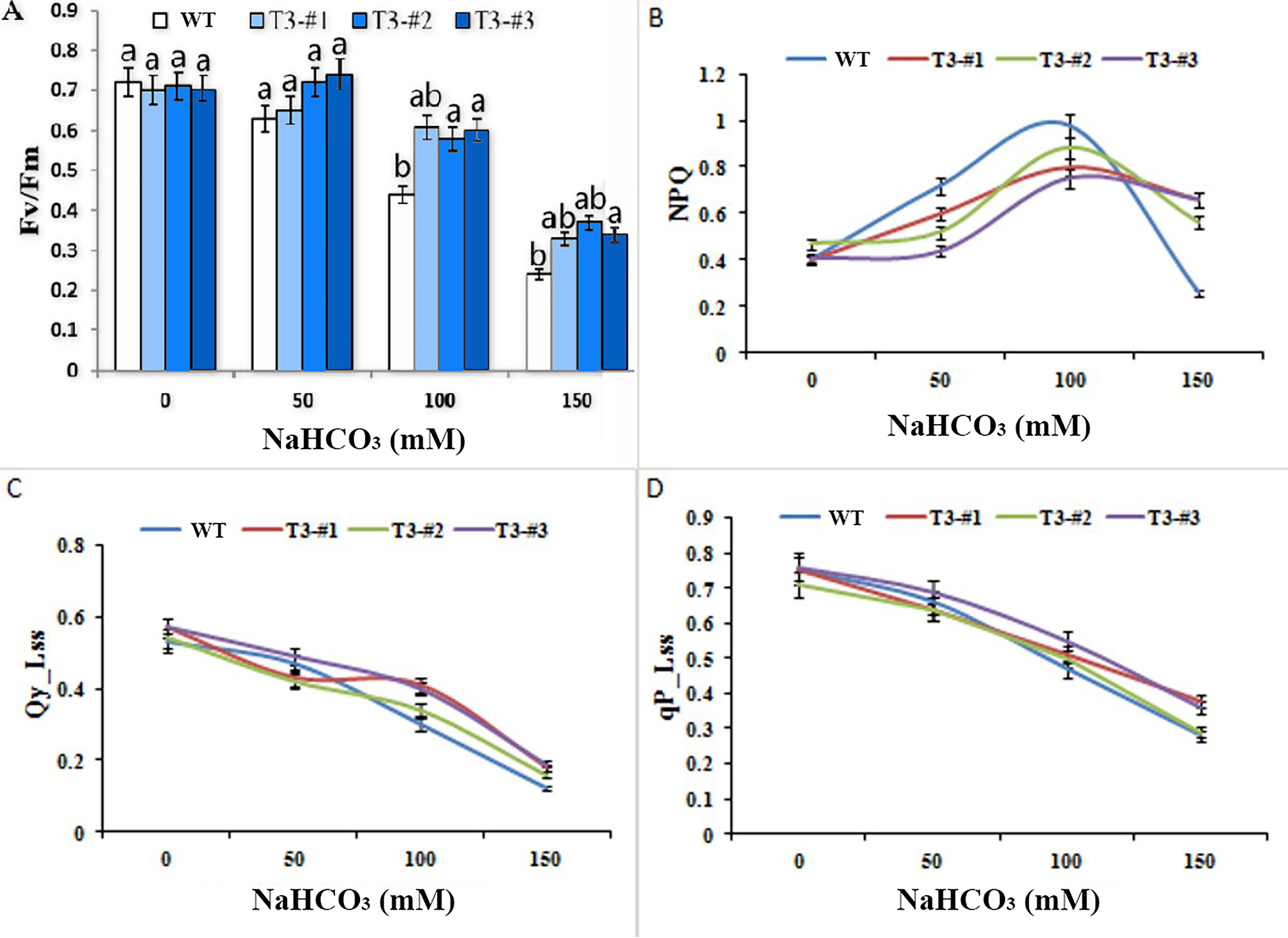

Supplement: Supplementary file 1 [file ijms-23-15686-s001.zip › S5.jpg]

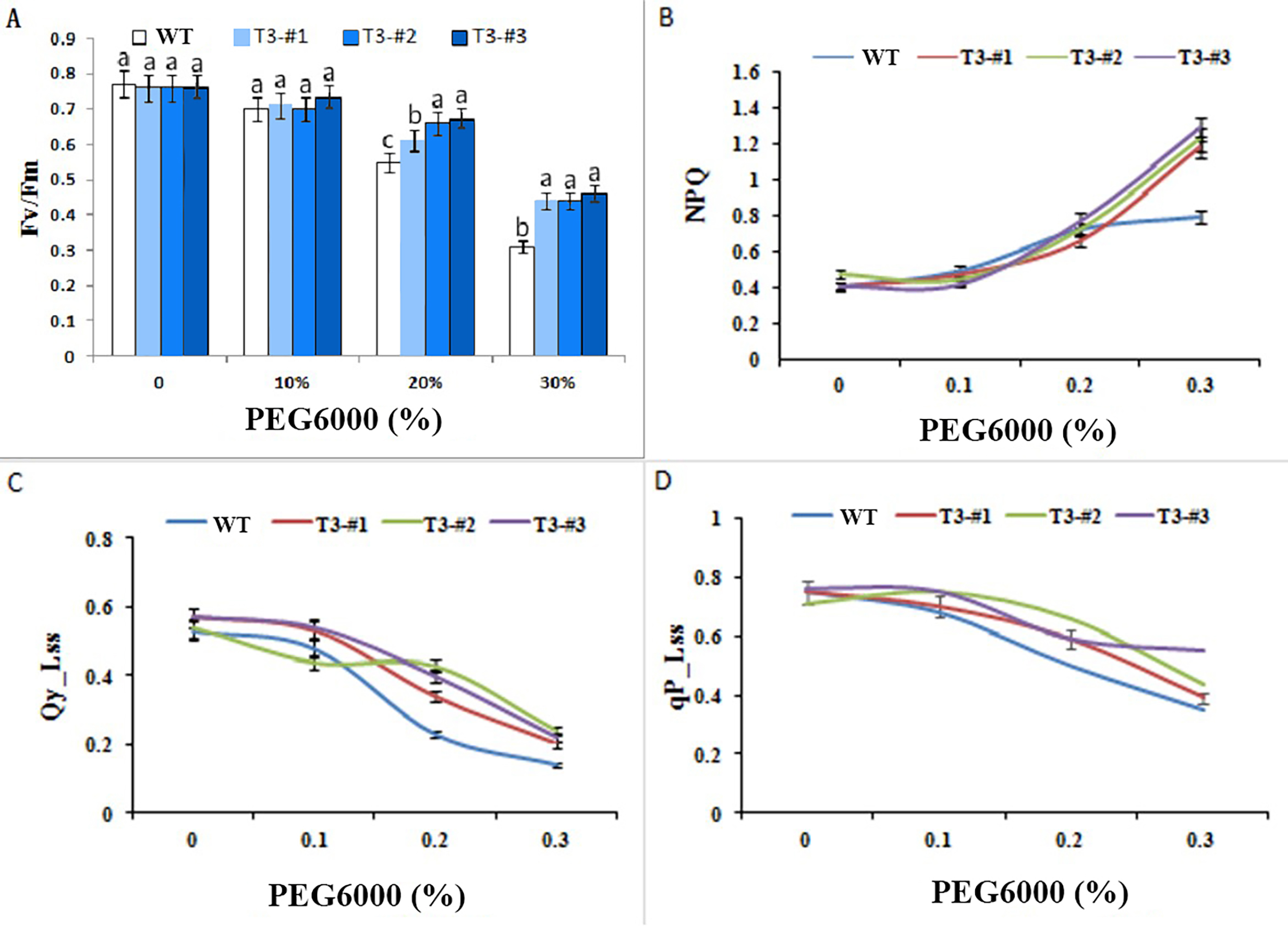

Supplement: Supplementary file 1 [file ijms-23-15686-s001.zip › S6.jpg]
